# Hepatopoietin Cn (HPPCn) Generates Protective Effects on Acute Liver Injury

**DOI:** 10.3389/fphar.2019.00646

**Published:** 2019-07-04

**Authors:** Na Li, Feng-Jiao Liu, Dan-Dan Li, Chun-Xia Sun, Jian Li, Mei-Hua Qu, Chun-Ping Cui, Da-Jin Zhang

**Affiliations:** ^1^School of Pharmacy, Key Laboratory of Applied Pharmacology, Weifang Medical University, Wei Fang, China; ^2^Center for Basic Medical Sciences, Sixth Medical Center of PLA General Hospital, Beijing, China; ^3^State Key Laboratory of Proteomics, National Center of Protein Sciences, Beijing Institute of Life Omics, Beijing, China

**Keywords:** hepatopoietin Cn, acute liver injury, liver regeneration, proliferation, protection

## Abstract

**Objective:** To observe the protective role of hapatopoietin Cn (HPPcn) on acute liver injury.

**Methods:** Six hours after 10 mmol/L CCl_4_, 150 mmol/L ethanol, or 0.6 mmol/L H_2_O_2_ treatment, SMMC7721 human hepatoma cells were incubated with 10, 100, or 200 ng/ml recombinant human HPPCn protein (rhHPPCn) for an additional 24 h. The cell survival rate was analyzed using the CCK-8 assay. The CCl_4_-induced apoptosis of SMMC7721 cells was detected by flow cytometry. Then, the levels of glutamic oxaloacetic transaminase (GOT), glutamic-pyruvic transaminase (GPT), malondialdehyde (MDA), lactate dehydrogenase (LDH), glutathione peroxidase (GSH-PX), and superoxide dismutase (SOD) in SMMC7721 cell lysates and cell culture supernatant were detected. SMMC7721 cells were treated with different concentrations of rhHPPCn (0, 10, and 100 ng/ml). The cell proliferation indexes (BrdU incorporation and PCNA expression) were detected by immunohistochemistry (IHC). An acute liver injury mouse model was established by a one-time intraperitoneal injection of 20% CCl_4_ at a volume of 5 ml/kg body weight. One hour after CCl_4_ injection, 1.25 or 2.5 mg rhHPPCn/12 h/kg body weight was injected *via* the tail vein. The serum levels of GOT and GPT were detected at different time points. Pathological changes in the liver were evaluated. PCNA expression levels were observed by IHC.

**Results:** rhHPPCn increased the survival rate of SMMC7721 cells and inhibited chemical toxicity-induced cell apoptosis. The levels of GOT, GPT, MDA, and LDH in the cell supernatant were significantly reduced, while GSH-PX and SOD were significantly increased after rhHPPCn treatment in the CCl_4_-treated SMMC7721 cells. BrdU incorporation and PCNA expression increased in a concentration-dependent manner, indicating that rhHPPCn promotes cell proliferation. The results showed that rhHPPCn significantly reduced the serum levels of GOT and GPT in CCl_4_-induced acute liver injury mice. rhHPPCn alleviated the tissue damage and increased PCNA expression, indicating the promotion of proliferation after acute injury.

**Conclusion:** rhHPPCn protects hepatocytes from chemical toxins by promoting proliferation and inhibiting apoptosis *in vivo* and *in vitro*. Our study provides new insights for the clinical treatment of acute liver injury.

## Introduction

Hepatopoietin Cn (HPPCn) is a growth factor that initially isolated from the hepatic stimulator substance (HSS) of newborn calf, which promotes hepatocyte proliferation. Sequence analysis showed that HPPCn belongs to the ANP32 (acidic/leucine-rich nuclear phosphoprotein 32) family (Tzirogiannis et al., 2005; Cui et al., 2008). ANP32 family members are involved in various biological processes, such as cell proliferation, differentiation, and apoptosis, and are related to tumorigenesis and drug resistance (Huyton and Wolberger, 2007). HPPCn is the only known member of the ANP32 family that can perform the role of cytokines outside the cell (Chang et al., 2010). It was reported that recombinant human HPPCn protein (rhHPPCn) can stimulate hepatocyte proliferation *in vitro* and *in vivo* by activating signaling pathways that include sphingosine kinase-1 and extra cellular signal-regulated kinase (Chang et al., 2010; Liu et al., 2013).

Acute liver injury is a common disease with various causes; 40% of the cases are drug-induced liver dysfunction, which requires a complex liver regeneration process for recovery (Bhushan and Apte, 2019). The liver regeneration process involves many liver-stimulating factors, growth factors, cytokines, and their downstream signaling pathways (Friederike et al., 2010). Most of the factors target a variety of cells and organs, lacking specificity for the liver regeneration process. However, rhHPPCn is a well-defined hepatocyte growth factor with specific hepatic-stimulating activities in partially hepatectomized (PH) mice (Cui et al., 2008; Liu et al., 2013). Studies have shown that rhHPPCn has no significant effect on various tissues in normal mice, including the liver. However, HPPCn expression is significantly increased in the liver of PH mice and rhHPPCn can stimulate DNA replication and Erk phosphorylation in hepatocytes of PH mice (Cui et al., 2008).

Our previous studies have shown that rhHPPCn and its family members can significantly reduce the damage of aflatoxin and alcohol on hepatocytes and promote hepatocyte proliferation (Chang et al., 2010; Liu et al., 2013). In addition, rhHPPCn can delay alcohol- or carbon tetrachloride (CCl_4_)-induced chronic liver fibrosis (Cui et al., 2010; Liu et al., 2013). Therefore, we hypothesized that rhHPPCn may play an important role in the protection against acute liver injury. This study used CCl_4_, ethanol, and hydrogen peroxide (H_2_O_2_) to induce a damaged hepatic cell model and CCl_4_ alone to induce an acute liver injury mouse model, and we observed the protective effects of rhHPPCn on acute liver injury to discover its underlying mechanisms.

## Materials and Methods

### Chemicals

The rhHPPCn protein was obtained by purification with Nickel Ion Column Affinity Chromatography from high cell density cultivation of the *Escherichia coli* strain BL21 (DE3)/p Cold II-HPPCn. Toxic substances, including ethanol (C_2_H_5_OH), H_2_O_2_, and CCl_4_, were purchased from Sionphrm Co. (Beijing, China).

### Cell Model

Human hepatoma SMMC7721 cells were obtained from Chinese Academy of Sciences Cell Bank (Shanghai, China), and cultured in RPMI1640 (Gibco, Life Technologies, CA, USA) supplemented with 10% fetal bovine serum (Hyclone, UT, USA), 100 IU/ml penicillin, and 100 mg/ml streptomycin (Hyclone, UT, USA). Cells were maintained in a humidified incubator at 37°C (Thermo Fisher Scientific, MA, USA) with 5% CO_2_.

### Animal Model of Acute Liver Injury With CCl_4_

To study the effect of rhHPPCn in acute liver injury, male Balb/c mice (20–22 g) were randomly grouped (eight in each group, n = 8). A single intraperitoneal injection of 20% CCl_4_ (in olive oil) in a volume of 5 ml/kg body weight was used to establish acute liver injury in the mice model. An equal volume of olive oil was intraperitoneally injected in Balb/c mice to establish the control group.

### Cell Viability by CCK-8 Assay After Toxic Damage and rhHPPCn Protein Stimulation

SMMC7721 cells were digested by 0.05% trypsin (containing 0.02% EDTA), then seeded in the 96-well culture plate at a concentration of 5 × 10^3^/well and incubated at 37°C for 24 h in a cell culture incubator. Then, a final concentration of 150 mmol/L ethanol, 0.6 mmol/L H_2_O_2_, or 10 mmol/L of CCl_4_ (1‰ DMSO preparation) was added and incubated for another 6 h. Different concentrations of rhHPPCn (final concentration of 10, 100, and 200 ng/ml) were added to the treated cells and cultured for another 24 h. A total of 10 μl of cell counting kit-8 solution (CCK-8, Dojindo Biotechnology, Kumamotoi, Japan) was added to each well, and the plates were incubated for 1 h at 37°C in a humidified incubator. The absorbance at 450 nm was measured using a microplate reader (Bio-Rad, Hercules, CA, USA).

### Cell Proliferation Observation

SMMC7721 cells in logarithmic growth phase were seeded in the 96-well culture plate at a concentration of 5 × 10^3^/well and incubated overnight. After the cells became adherent, 10 mmol/L of CCl_4_ (1‰ DMSO preparation) was added and incubated for 6 h. rhHPPCn with a final concentration of 0, 10, and 100 ng/ml was added to cells and incubated for 48 h. BrdU (10 μmol/L) was added and incubated for 2 h before cell harvesting. The cells were fixed with 4% paraformaldehyde and perforated with 0.3% TritionX-100 for 20 min on ice. Endogenous peroxidase was removed by 3% H_2_O_2_ for 30 min, and the samples were blocked with 10% FBS for 30 min. BrdU (1:40, rabbit mAb, ab6326, Abcam, Cambridge, MA, USA) and PCNA (1:100, rabbit mAb, ab92552, Abcam, Cambridge, MA, USA) antibodies were added and incubated overnight at 4°C. Secondary antibodies (goat anti-rabbit, Zsbio) labeled with HRP were added the next day. The results were visualized with DAB and observed under a microscope (IX71, Olympus, Japan).

### Apoptosis Detection

The cells were treated in the same manner as above and then inoculated into six-well plates with 3.6 × 10^5^ cells per well. The cells were divided into four groups: the vehicle control group; the rhHPPCn group (100 ng/ml rhHPPCn); the CCl_4_ group (10 mmol/L CCl_4_); and the CCl_4_+rhHPPCn group (10 mmol/L CCl_4_ and 100 ng/ml rhHPPCn). The cells were cultured overnight. The CCl_4_+rhHPPCn group and the CCl_4_ group were treated with 10 mmol/L CCl_4_ for 6 h; the CCl_4_+rhHPPCn group was stimulated with 100 ng/ml rhHPPCn for 24 h. Flow cytometry with Annexin V was used for the detection of cell apoptosis. Apoptotic cells were detected using Annexin V and propidium iodide (PI). In detail, the cells were harvested and washed twice in cold PBS. The cell pellets were then resuspended in 1×Annexin V-binding buffer containing 10 mmol/L 4-(2-hydroxyethyl)-1-piperazineethanesulfonic acid (HEPES), 140 mmol/L NaCl, and 5 mmol/L CaCl_2_ at a concentration of 1 × 10^5^ cells/ml. The suspension (100 μl containing 1 × 10^5^ cells), 5 μl of Annexin V-FITC, and 10 μl of PI were added into a 5-ml culture tube. The tube was gently vibrated and incubated for 15 min in the dark at room temperature. After a binding buffer (400 μl) was added to the tube, the cells were analyzed by flow cytometry.

### Detection of Cytotoxicity Relevant Indicators in SMMC7721 Cells

CCl_4_-induced cell injury was performed in the same manner as described. After 6 h of CCl_4_ treatment, rhHPPCn with a final concentration of 10, 100, or 200 ng/ml was added to the cells. Three duplicate wells were established in the experiment. After 24 h, glutamic oxaloacetic transaminase (GOT), glutamic-pyruvic transaminase (GPT), and superoxide dismutase (SOD) levels were detected after cell disruption using commercially available kits (Njjcbio, Nanjing, China). Glutathione peroxidase (GSH-PX), lactate dehydrogenase (LDH), and malondialdehyde (MDA) levels in cell supernatant were measured using commercial assay kits (Njjcbio, Nanjing, China).

### Observation of Serum GOT and GPT Levels of the Acute Liver Injury Mice Treated by rhHPPCn

To determine whether the protection of rhHPPCn against acute liver injury is dose-dependent, gradient concentration of rhHPPCn at 1, 5, 15, 25, and 50 μg of rhHPPCn/12 h/20 g body weight was injected by tail vein at 1 h after CCl_4_ injection. PBS injection was used as a model control. The animals were sacrificed at 24 h after rhHPPCn injection. Blood was collected through the medial canthus vein of each mouse. The serum GPT and GOT levels were measured using ELISA kits (Njjcbio, Nanjing, China). To determine the time course of the effect of rhHPPCn on acute liver injury, 50 μg of rhHPPCn/12 h/20 g body weight was injected *via* tail vein 1 h after the model establishing. Blood was collected at 0, 12, 24, 36, and 48 h after rhHPPCn treatment. Serum level of GPT and GOT was detected by ELISA kits (Njjcbio, Nanjing, China).

### The Effect of HPPCn on PCNA Expression in the Liver of Acute Liver Injury Mice

Acute liver injury model mice were tail-vein injected rhHPPCn (1.25, 2.5 mg/12 h/kg body weight) 1 h after CCl_4_ treatment. PBS injection was used as a model control. Mice were sacrificed 24 h after rhHPPCn treatment. The liver tissues were fixed with 4% paraformaldehyde to prepare wax blocks. The liver specimens were dewaxed in xylene, rehydrated in ethanol, and washed with PBST for 10 min, and hematoxylin and eosin (H&E) staining was performed. IHC analysis was performed using tissue sections. Liver specimens were treated with 3% hydrogen peroxide for 30 min and blocked with 10% FBS for 30 min; then, the tissue slices were incubated with anti-PCNA antibodies (1:100, ab92552, Abcam, Cambridge, MA, USA). The tissue slices were incubated with HRP-conjugated secondary antibody for 1 h at room temperature. Nonimmune rabbit serum was used for the negative controls. After rinsing with PBST three times for 5 min each, the slices were incubated with diaminobenzidine (DAB) for 10 min at room temperature. The location and level of protein expression were observed and analyzed under a microscope (IX71, Olympus, Japan).

### Statistical Analysis

All experiments were performed at least thrice. Quantitative data are presented as the mean ± standard deviation (SD). Experimental mice were randomly separated into seven groups (eight per group, n = 8). Comparisons between two groups were made by Student’s test one-factor ANOVA for repeated measurements followed by Tukey’s test for pairwise comparison of the experimental groups. All tests were performed using the SPSS 11.5 (SPSS, Inc., Chicago, IL, USA), and two-tailed P values of less than 0.05 were considered significant.

## Results

### rhHPPCn Increased the Survival of SMMC7721 Cells Injured by Toxic Substances

**Figure 1:**
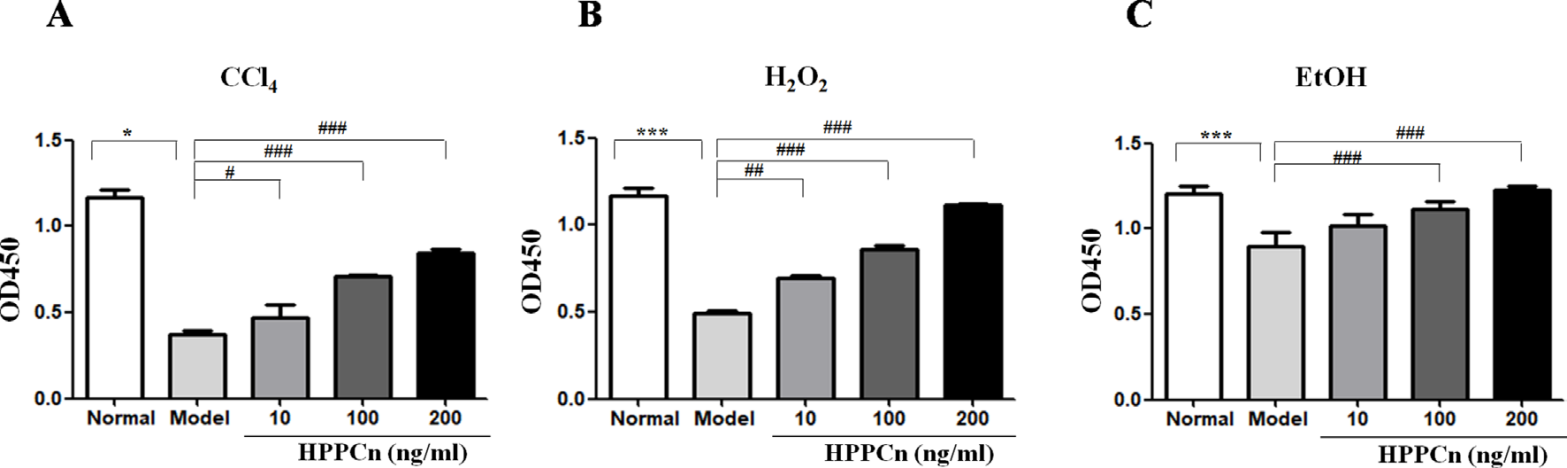
rhHPPCn increased the survival of SMMC7721 cells injured by toxic substances. A CCK8 assay was used to evaluate the protective effect of rhHPPCn on SMMC7721 cells injured by different toxic chemicals. **(A)** Treated with different concentrations of rhHPPCn for 24 h after 10 mmol/L CCl_4_ injury. **(B)** Treated with different concentrations of rhHPPCn for 24 h after 0.6 mmol/L H_2_O_2_ injury. **(C)** Treated with different concentrations of rhHPPCn for 24 h after 150 mmol/L ethanol injury. *P < 0.05, ***P < 0.001 *vs.* Control; ^#^ *P* < 0.05, ^##^ *P* < 0.01, ^###^ *P* < 0.001 *vs.* Model.

The SMMC7721 cell viability after a 6-h treatment of CCl_4_, ethanol, or H_2_O_2_ was detected by CCK-8 assay. As shown in **Figure 1A**, cell viability was significantly reduced after a 6-h treatment with CC1_4_. After another 24-h incubation with rhHPPCn at a concentration of 10, 100, or 200 ng/ml, the survival rate of cells in the rhHPPCn-treated group increased 1.25, 1.89, and 2.26 times, respectively, compared with the CCl_4_-treated model cells (**Figure 1A**). The protection effect of rhHPPCn from ethanol and H_2_O_2_ in SMMC7721 cells displayed a similar pattern at that in CCl_4_-treated SMMC7721 cells (**Figures 1B, C**).

### rhHPPCn Promotes SMMC7721 Cell Proliferation

**Figure 2:**
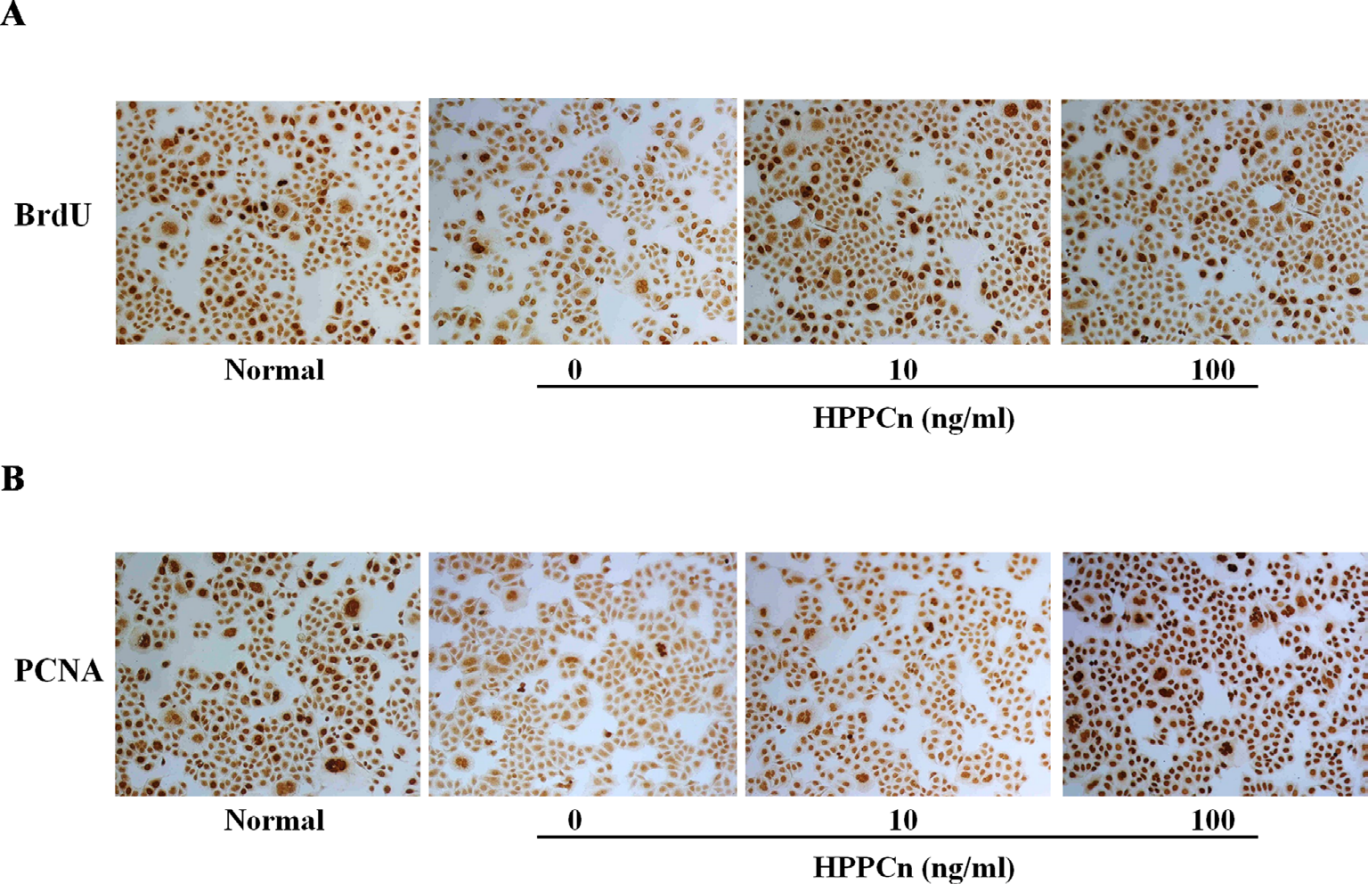
Effects of rhHPPCn on the proliferation of SMMC7721 cells treated with CCl_4_ (100×). The BrdU incorporation and the PCNA expression in SMMC7721 cells treated with different concentrations of rhHPPCn after 10 mmol/L CCl_4_ injury detected by IHC. **(A)** The incorporation of BrdU in SMMC7721 cells treated with different concentrations of rhHPPCn after 10 mmol/L CCl_4_ injury. **(B)** The expression of PCNA in SMMC7721 cells treated with different concentrations of rhHPPCn after 10 mmol/L CCl_4_ injury.

rhHPPCn significantly promoted SMMC7721 cell proliferation. The results showed that rhHPPCn significantly increased BrdU incorporation in CCl_4_-treated SMMC7721 cells in a concentration-dependent manner (**Figure 2A**). The results suggested that rhHPPCn could promote DNA synthesis in SMMC7721 cells after CCl_4_-induced acute injury. **Figure 2B** showed that rhHPPCn increased PCNA expression in CCl_4_-induced acute injury SMMC7721 cells in a concentration-dependent manner. The results suggested that rhHPPCn could also promote the proliferation of SMMC7721 cells after acute injury.

### rhHPPCn Inhibits SMMC7721 Cell Apoptosis

**Figure 3:**
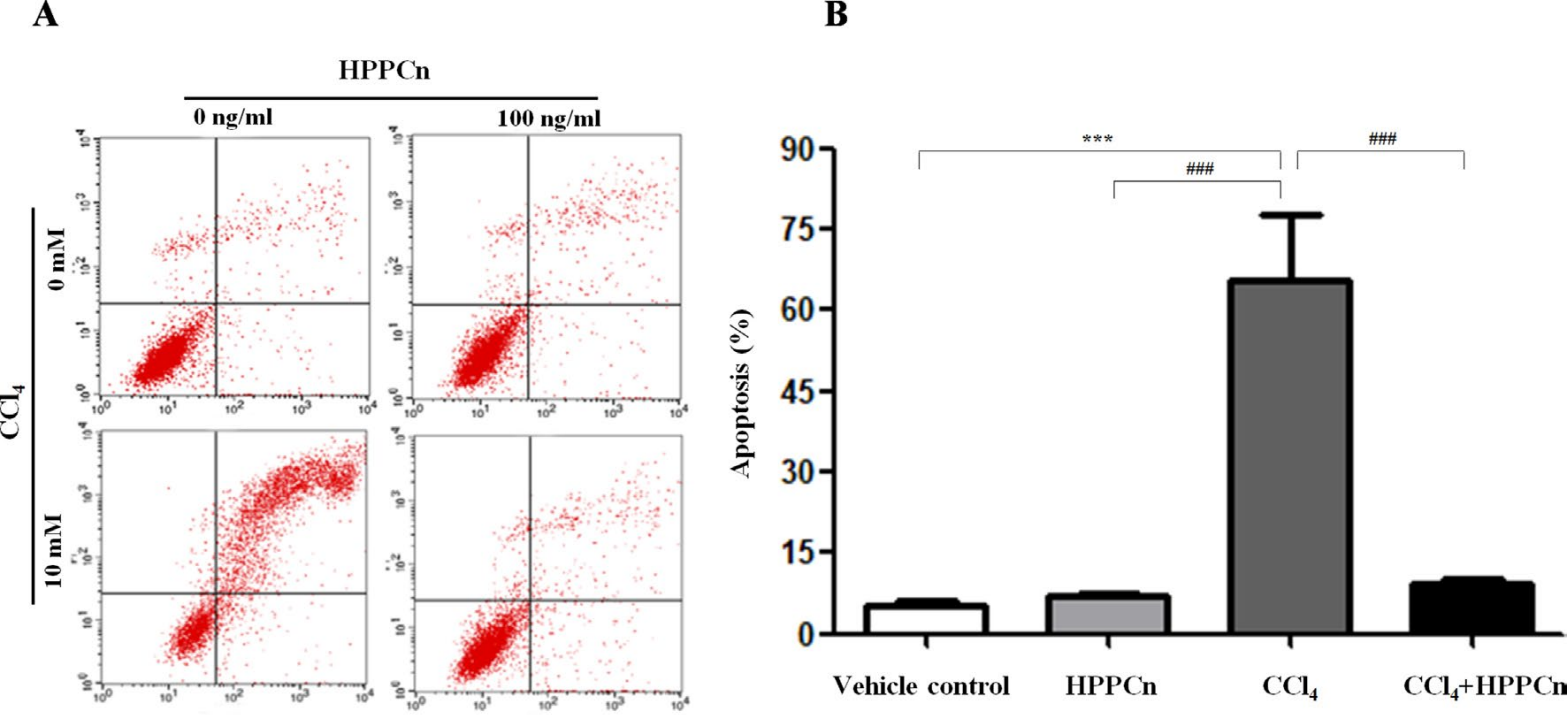
rhHPPCn inhibits the apoptosis of SMMC7721 cells treated by CCl_4_. The cell apoptosis was detected using FITC-conjugated Annexin V and PI by flow cytometry. **(A)** Image of vehicle control group, HPPCn group (100 ng/ml HPPCn), CCl_4 _group (10 mmol/L CCl_4_), and CCl_4_+HPPCn group (10 mmol/L CCl_4_, 100 ng/ml rhHPPCn). **(B)** Statistical analysis of the apoptosis rate of the four groups (vehicle control group, HPPCn only group, CCl_4 _only group, and CCl_4_+HPPCn group). **P < 0.01, ***P < 0.001 *vs.* the vehicle control group; ^###^ *P* < 0.001 *vs.* the CCl_4_ group.

It has been demonstrated that CCl_4_ exposure is linked to hepatocyte apoptosis, and rhHPPCn can reverse the apoptosis caused by this chemical injury. CCl_4_-induced apoptosis was significantly inhibited in cells treated with 100 ng/ml rhHPPCn (**Figure 3A**). Without CCl_4_-induced injury, the cell apoptosis rate in the rhHPPCn group showed no significant difference compared with the vehicle control group (6.95 ± 0.67% *vs.* 5.39 ± 0.86%), indicating that rhHPPCn has no cytotoxic effect. In the CCl_4_ group, 65.59 ± 12.30% of SMMC7721 cells were apoptotic, while the apoptosis rate of the CCl_4_+rhHPPCn group decreased to 9.18 ± 1.04% (**Figure 3B**). The results showed the protection effect of rhHPPCn in CCl_4_-induced hepatocytes apoptosis.

### rhHPPCn Protected SMMC7721 Cells From Chemical Toxicants

**Figure 4:**
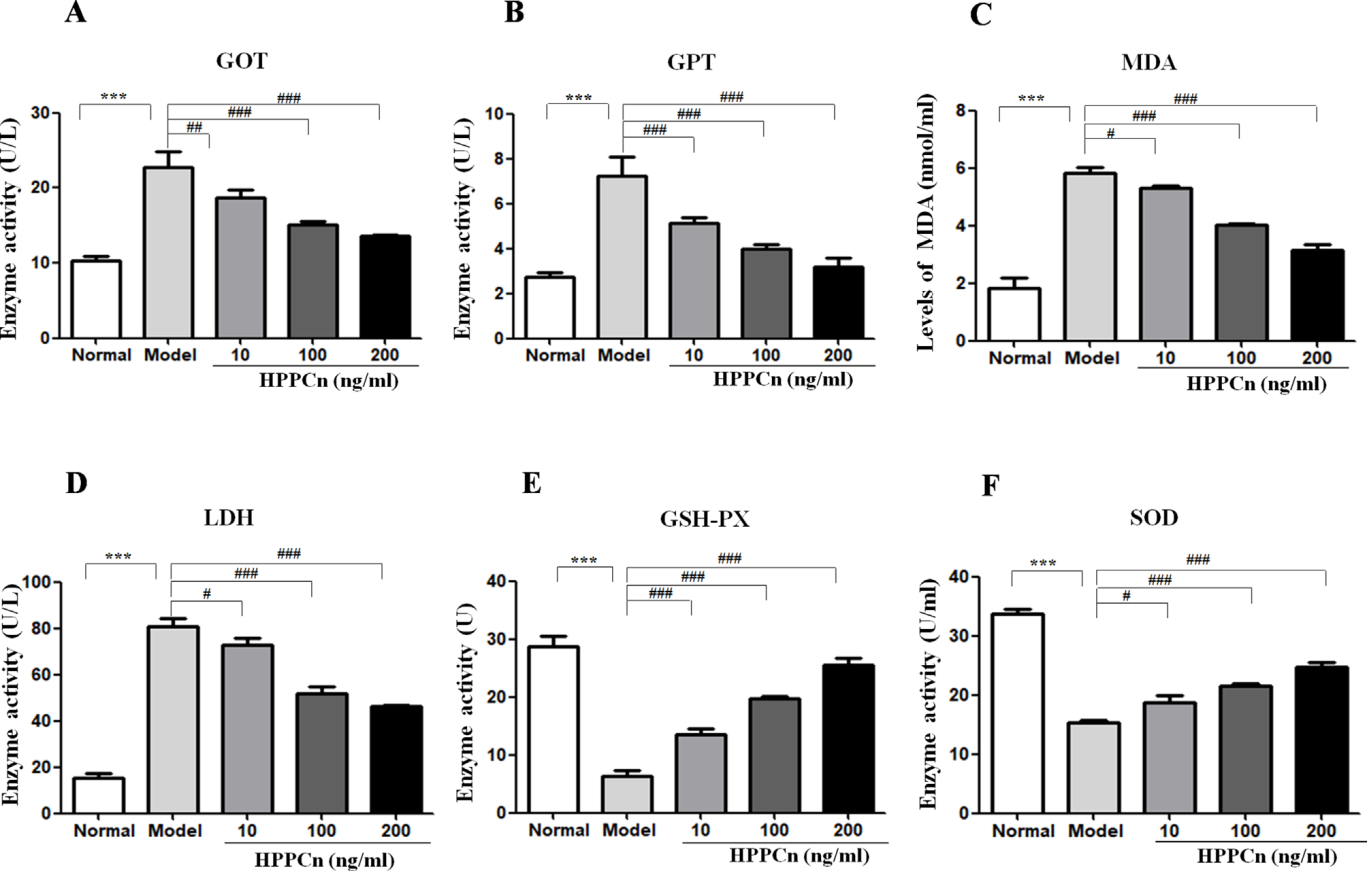
rhHPPCn reduced CCl_4_-induced cytotoxicity in SMMC7721 cells. The levels of hepatocyte injury indicators, including GOT, GPT, MDA, LDH, GSH-PX, and SOD in the cell lysate were evaluated by ELISAs in cells treated with different concentrations of rhHPPCn for 24 h after 10 mmol/L CCl_4_-induced cell injury. **(A–D)** GOT, GPT, MDA, and LDH. **(E** and **F)** GSH-PX, SOD. rhHPPCn treatment significantly reversed the increased serum level of GOT, GPT, MDA, and LDH and significantly reversed the decreased serum level of GSH-PX and SOD in a dose-dependent manner after acute cell injury. ***P < 0.001 *vs.* Control; ^#^ *P* < 0.05, ^##^ *P* < 0.01, ^###^ *P* < 0.001 *vs.* Model.

CCl_4_ at 10 mmol/L was found to be cytotoxic in SMMC7721 cells, with elevated levels of GOT, GPT, LDH, and MDA and decreased levels of GSH-PX and SOD. After treatment with rhHPPCn, the levels of the damage indicators LDH, MDA, GOT, and GPT were decreased, while the levels of the protective indicators GSH-PX and SOD were increased in CCl_4_-damaged SMMC7721 cells in a dose-dependent manner (**Figure 4**).

### rhHPPCn Protected CCl_4_-Induced Acute Liver Injury Mice in a Concentration- and Time Course-Dependent Manner

**Figure 5:**
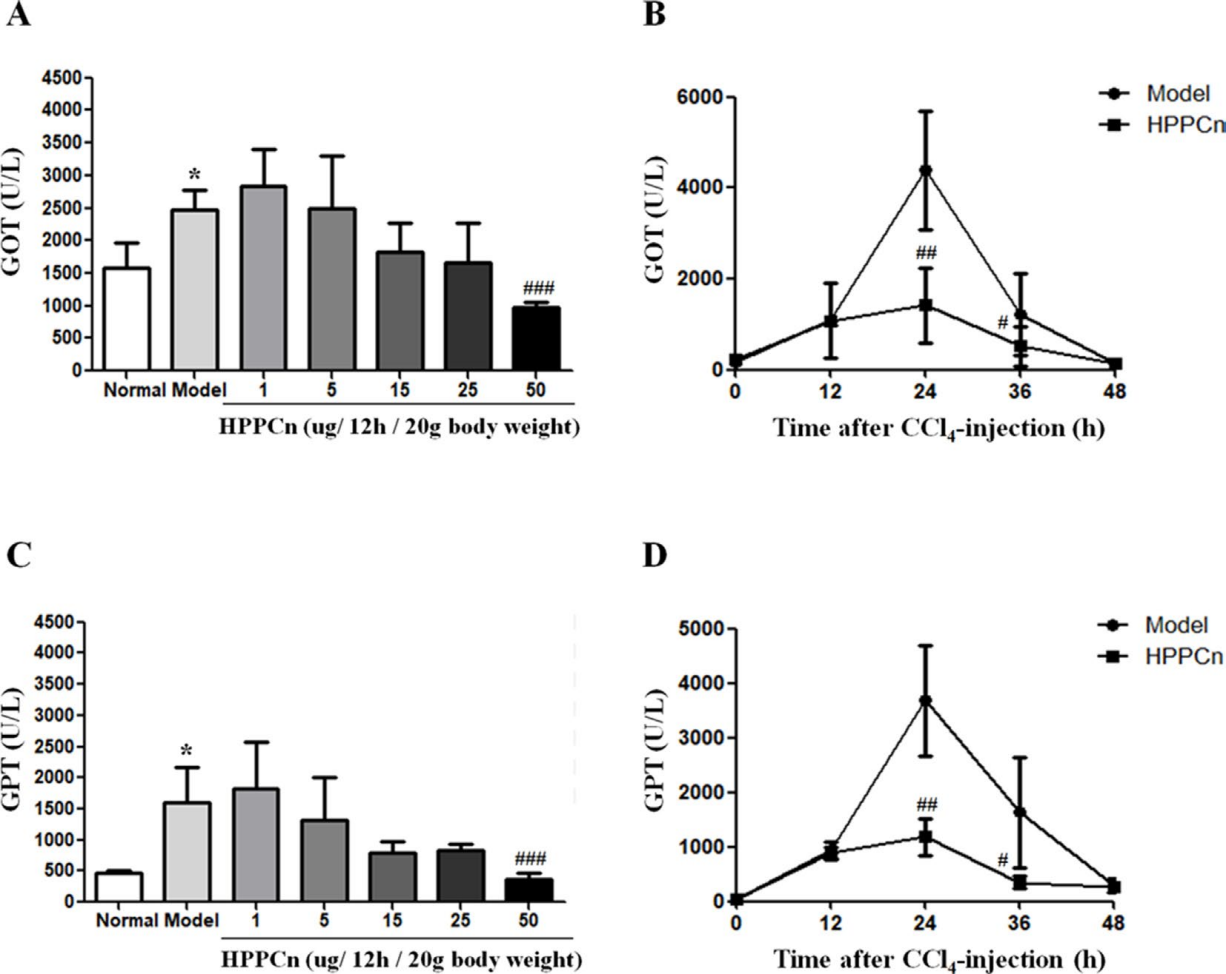
The effects of rhHPPCn on the serum levels of GOT and GPT in CCl_4_-induced acute liver injury model mice. **(A** and **C)** The serum levels of GOT and GPT in CCl_4_-induced acute liver injury model mice treated with different concentrations of rhHPPCn for 24 were evaluated by ELISA kits. **(B** and **D)** The serum levels of GOT and GPT in CCl_4_-induced acute liver injury model mice treated with rhHPPCn for different time periods. The results showed that rhHPPCn could decrease the levels of GOT and GPT in the serum of acute liver injury model mice in a concentration- and time-dependent manner. *P < 0.05 *vs.* Control; ^#^ *P* < 0.05, ^##^ *P* < 0.01, ^###^ *P* < 0.001 *vs.* Model.

Mice with CCl_4_-induced acute liver injury were treated with different concentrations of rhHPPCn. After 24 h, serum GPT and GOT levels were detected. The results showed that rhHPPCn could reduce serum GPT and GOT levels in mice with CCl_4_-induced acute liver injury (**Figure 5A**). When the dose of rhHPPCn reached 50 μg/kg, there was a significant difference compared with the model group. Therefore, the effect of the protective effect of 50 μg/kg rhHPPCn on CCl_4_-induced acute liver injury in a time-dependent manner was explored. The results showed that the levels of GPT and GOT in mice increased rapidly after CCl_4_ treatment, peaked at 24 h, and remained at high level till 36 h; however, these levels returned to normal after 48 h CCl_4_ treatment (**Figure 5B**). This finding indicated that the peak of injury was within 48 h of acute injury, and the protective effect at this stage was most important. Compared with the model group, the serum GPT and GOT levels of mice were significantly decreased by more than three times at 24 and 36 h after CCl_4_-induced acute liver injury. After mice with CCl_4_-induced acute liver injury were treated with rhHPPCn, the serum GPT and GOT levels were significantly decreased at 24 and 36 h. The results indicated that rhHPPCn had a protective effect on CCl_4_-induced acute liver injury in mice (**Figure 5**).

### rhHPPCn Reduces CCl_4_-Induced Liver Injury in Mice

**Figure 6:**
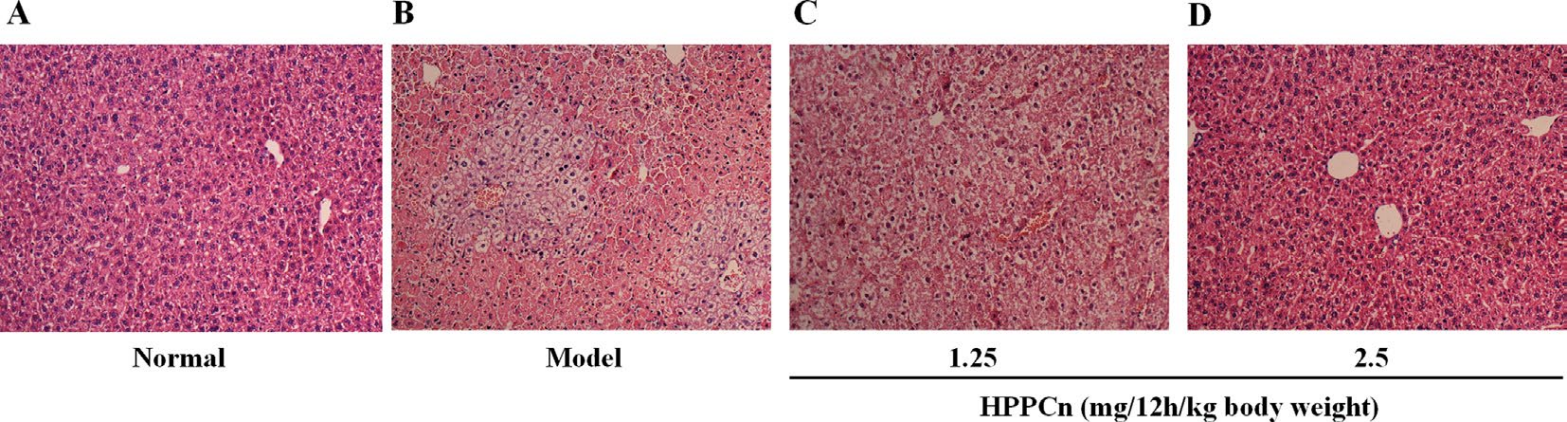
rhHPPCn reduced the acute liver injury detected by HE staining. **(A)** Control normal mice. **(B)** Acute liver injury model mice. **(C)** Acute liver injury model mice treated with rhHPPCn at 1.25 mg/12 h/kg body weight for 24 h. **(D)** Acute liver injury model mice treated with rhHPPCn at 2.5 mg/12 h/kg body weight for 24 h.

As shown in **Figure 6**, the livers of mice treated with CCl_4_ for 24 h showed hepatocyte edema, vacuolization, necrosis, and structural changes compared to normal mice (**Figures 6A, B**). The CCl_4_-induced liver injury was significantly reduced in mice after 24 hs treatment of rhHPPCn at 1.25 mg/12 h/kg or 2.5 mg/12 h/kg body weight in a dose-dependent manner (**Figures 6C, D**). The HE staining of 2.5 mg/12 h/kg rhHPPCn group mice liver showed neatly arranged lobules structure and hepatocytes, and clear and complete nuclei of hepatocytes, similar to that in the normal group. The results indicated that rhHPPCn could obviously protect liver form acute injury by CCl_4_.

### HPPCn Promotes Hepatocyte Proliferation in CCl_4_-Induced Acute Liver Injury Mice

**Figure 7:**
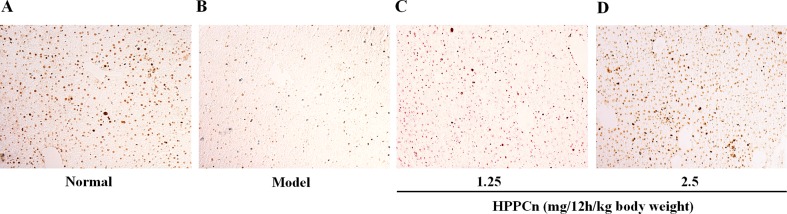
rhHPPCn promoted hepatocyte proliferation in CCl_4_-induced acute liver injury model mice. PCNA expression was detected by immunohistochemistry after acute injured liver treated with different concentrations of rhHPPCn. **(A)** Control normal mice. **(B)** Acute liver injury model mice. **(C)** Acute liver injury model mice treated by rhHPPCn at 1.25 mg/12 h/kg body weight for 24 h. **(D)** Acute liver injury model mice treated by rhHPPCn at 2.5 mg/1 2h/kg body weight for 24 h.

Different doses of rhHPPCn (1.25, 2.5 mg/12h/kg body weight) were administrated at 1 h after CCl_4_ induced acute liver injury. IHC results showed that PCNA expression was significantly reduced in the CCl4-induced acute injury compared with the control group (**Figures 7A, B**). Furthermore, compared with the model group, treatment with rhHPPCn protein significantly promoted PCNA expression in the CCl_4_-induced injured liver in a dose-dependent manner (**Figures 7C, D**).

## Discussion

The liver is an important body organ because it participates in a variety of metabolic functions, such as de-oxidation, glycogen storage, and the synthesis of secreted proteins. Liver injury (induced by toxins, viruses, surgery, etc.) forces the surviving hepatocytes to perform their physiological functions and restore liver volume through liver regeneration. The mechanism of liver injury varies with etiology. Necrosis, apoptosis, and the impaired regeneration ability of hepatocytes are the three main presentations of hepatocytes after the liver injury (Bechmann et al., 2010). In this study, the CCK-8 assay and H&E staining were used to observe hepatocyte survival and necrosis, and flow cytometry was used to observe hepatocyte apoptosis. BrdU incorporation and PCNA expression were investigated to determine the proliferative ability of hepatocytes after CCl_4_-induced acute liver injury.

Hepatic regulatory factors, including rhHPPCn, play an important role in the liver regeneration process (Jansen et al., 1990; De et al., 2008; De et al., 2011). The survival of a patient with more than 80% of hepatocyte damage depends entirely on liver regeneration. However, liver regeneration is often insufficient in severe liver disease. A high mortality rate is often found in fulminant hepatitis patients with a hepatocyte volume of less than 12%. Therefore, finding specific and safe liver-stimulating factors is of great significance for the treatment of acute liver injuries.

Many cytokines involved in liver regeneration also demonstrate protective effects against different liver damages. HGF is an effective DNA synthesis stimulator in hepatocytes and also protects the liver against fibrosis and other types of damage (Imai et al., 2003; Toshimi et al., 2010; Huard et al., 2012). HSS protects the liver from the hepatic agent CCl_4_ and galactosamine against fibrosis (Gao and Zhou, 2005; Li et al., 2011). The liver regeneration enhancer ALR can inhibit liver atrophy induced by Eck’s fistula (portal vein and inferior vena cava anastomosis) (Rosemurgy et al., 2002; Francavilla et al., 2010; Zeng et al., 2010; Xi et al., 2013). The results of the study showed that these factors can effectively protect the liver from further damage *in vivo* and improve animal survival by promoting hepatocyte DNA synthesis and liver regeneration. However, these hepatic regenerative cytokines are limited in their application due to a lack of specificity composition and mechanism.

LaBrecque and Pesch (1975) reported that HSS is only present in the liver of newborn mammals or regenerative liver, and it not only stimulates the DNA synthesis and mitosis of hepatocytes *in vitro* but also promotes liver regeneration in some liver-resected animals. It has no such effects on other organs, such as bone marrow, spleen, or kidney (Liatsos et al., 2003; Tzirogiannis et al., 2005).

The rhHPPCn was initially isolated and identified from the newborn calf liver HSS through the combination of traditional biochemical separation methods and newly developed proteomics research techniques (Cui et al., 2008). Studies have shown that rhHPPCn expression after partial hepatectomy in mice is temporally dynamic (Cui et al., 2008; Liu et al., 2013). Similar to HSS, rhHPPCn’s stimulating proliferative activity exhibits organ specificity. Not only does it stimulate hepatocytes cultured *in vitro* to initiate DNA synthesis and mitosis, it also promotes liver regeneration in some liver-resected animals *in vivo* with no effects on other organs.

CCl_4_ is a pro-hepatic toxicant and is considered a classic chemical inducer of acute liver injury in a model commonly used for screening for liver-protecting agents. In this study, CCl_4_ was used to induce an acute liver injury mouse model. Both *in vivo* and *in vitro* experiments showed that CCl_4_ caused liver tissue necrosis, decreased hepatocyte proliferation, and increased hepatocyte apoptosis, etc. This study showed that rhHPPCn significantly promoted DNA replication and the expression of proteins involved in proliferation. Our results also indicated that rhHPPCn could inhibit hepatocyte apoptosis and improve hepatocyte survival.

Previous studies have shown that HPPCn expression begins to increase at 2 h after partial hepatectomy, peaks at 12–24 h, and decreases slowly afterward (Cui et al., 2008). It was suggested that HPPCn might be related to the transition of hepatocytes from G1 phase to S phase in the early stage of liver regeneration, thus promoting hepatocytes to cross the restriction point of G1 phase and carry out DNA replication (Cui et al., 2008; Dechêne et al., 2010). These studies mainly focused on the function of HPPCn in chronic liver injury; however, the mechanism in chronic and acute liver injury varies. The current study focused on the protection function of HPPCn on acute liver injury caused by CCl_4_. This study is an in-depth supplement to the previous study.

CCl_4_ mainly destroys the hepatocyte membrane through the oxidative stress reaction caused by its free radical metabolites and increases the permeability of the cell membrane, thereby causing liver damage. After CCl_4_ enters the body, it is metabolized to a trichloromethyl group under the action of liver microsomal enzyme. The trichloromethyl group can destroy the structure of hepatocyte membrane and increase the permeability of the hepatocyte membrane, resulting in the release of intracellular enzymes GPT and GOT from the cell and an increase in GPT and GOT levels in the blood. Therefore, GPT and GOT in serum can directly reflect the degree of liver damage. This study established a model of acute liver injury induced by CCl_4_ in mice, and observed the protective effect of rhHPPCn on liver injury. The results showed that serum GPT and GOT levels increased significantly in the CCl_4_-induced acute liver injury model group, and rhHPPCn administration significantly reduced serum GPT and GOT levels in CCl_4_-induced acute liver injury mice in a concentration- and time-dependent manner. H&E staining of tissue sections showed that rhHPPCn significantly reduced necrosis and injury of hepatocytes, which indicates a protective effect of rhHPPCn on toxic chemical-induced liver injury.

CCl_4_ undergoes redox reactions in the body to generate a large number of free radicals, which attack the unsaturated fatty acids on the cell membrane and induce lipid peroxidation. LDH is a cytoplasmic enzyme, which is released when the cell membrane is damaged. The amount of LDH detected can be used as an indicator for determining the number of dead cells. MDA is one of the final products of lipid peroxidation, and it accumulates in the process of CCl_4_-induced liver injury. MDA then combines with biomacromolecules to form aldehyde, which further undermines the structure and function of the hepatocyte membrane. SOD, an effective metalloenzyme, can catalyze the disproportionation of superoxide anion to H_2_O_2_ and O_2_. GSH-PX catalyzes the reduction of toxic peroxides into non-toxic hydroxy compounds, while also reducing H_2_O_2_ and hydroperoxides to water, removing lipid hydrogen peroxide from the cell membrane, thereby terminating lipid peroxidation. The results of this study showed that rhHPPCn treatment effectively reduced the levels of MDA, LDH, GPT, and GOT in human hepatoma SMMC7721 cells after CCl_4_ treatment, and increased the levels of SOD and GSH-PX.

This study showed that rhHPPCn attenuated the toxicity of CCl_4_, ethanol, and H_2_O_2_ on SMMC7721 cells. rhHPPCn significantly increased the expression of PCNA in the liver of mice and SMMC7721 cells after CCl_4_ treatment and significantly inhibited the apoptosis of hepatocytes. These results indicate that rhHPPCn has a protective effect on acute liver injury, reducing toxic damage and protecting the liver. Its mechanism may be related to the involvement of rhHPPCn in regulating liver regeneration and inhibiting apoptosis.

Previous studies have shown that HPPCn can be secreted by non-canonical pathways. HPPCn may act as a cytokine in an autocrine or paracrine form to interact with receptors on the surface of hepatocytes, activating signaling pathways, such as SPK, Erk1/2, and Jak-Stat3, to promote hepatocyte proliferation and liver regeneration. For example, HPPCn significantly increases the phosphorylation of Erk1/2 in the liver of hepatectomized mice (Cui et al., 2008). It was reported that HPPCn attenuates oxidative injury and fibrosis induced by ethanol feeding and that the SphK1/S1P/S1PRs signaling pathway contributes to the protective effect of HPPCn on hepatocyte apoptosis and HSC activation (Liu et al., 2013). HPPCn activates signaling pathways involved in the survival of HCC cells and up-regulates myeloid cell leukemia-1 (Mcl-1) expression via the MAPK and SPK1 pathways (Chang et al., 2010; Liu et al., 2013).

Liver regeneration is a complex process involving multiple types of cells and factors. After liver tissue was partially resected or injured by toxic chemicals, various hepatic cells underwent proliferation, and liver regeneration was initiated. This procedure was first activated in residual hepatocytes with DNA synthesis at 10 h after liver injury. The weight and function of the liver were restored by hepatocyte proliferation. Non-parenchymal cells, such as stellate and Kuffer cells, were observed undergoing DNA synthesis starting at 24 h, and peaking at 48 h, after liver injury. This study mainly focused on the effect of HPPCn on hepatocytes in the early stage of liver injury. The effect of HPPCn on stellate, Kuffer cells, and fibroblasts, which play a role in the termination stage, has not been studied.

Although studies have suggested that HPPCn may be associated with tumorigenesis (Chang et al., 2010; Zhu et al., 2010), there is no direct evidence that HPPCn protein directly or indirectly causes a high risk of tumor generation. A large amount of experimental data indicate that extracellular administration of rhHPPCn has little proliferative effect on normal mammalian cells and tumor cells without toxicant injury. Since HPPCn protein is stable and is easy to prepare, the effect of short-term application of HPPCn to improve the prognosis in acute liver injury and reveal the underlying mechanism is worth exploring.

## Ethics Statement

This study was carried out in accordance with the recommendations of Weifang Medical University, China. The experimental protocol was approved by the Research Ethics Committee of Weifang Medical University, China.

## Author Contributions

D-JZ, M-HQ, and C-PC designed and led the study, and wrote the manuscript. NL, F-JL, D-DL, C-XS, JL, M-HQ, C-PC, and D-JZ performed the experiments and gathered the statistical analysis data. All co-authors commented on the manuscript and agreed with the manuscript results and conclusions.

## Funding

This work was funded by the National Natural Science Foundation of China (81472350, 81871892, 31071256, and 31671208), the Natural Science Foundation of Shandong Province (ZR2015HL128), and Technology Development Plan of Weifang (2018YX027).

## Conflict of Interest Statement

The authors declare that the research was conducted in the absence of any commercial or financial relationships that could be construed as a potential conflict of interest.
